# Complete Genome Sequence of Bacteriophage MA12, Which Infects both *Campylobacter jejuni* and *Salmonella enterica* Serovar Enteritidis

**DOI:** 10.1128/genomeA.00810-16

**Published:** 2016-12-08

**Authors:** Sunjin Lee, Taesoo Kwon, Su-Jin Chae, Jong-Hyun Kim, Yeon Ho Kang, Gyung Tae Chung, Dae-Won Kim, Deog-Yong Lee

**Affiliations:** a Division of Enteric Diseases, Center for Infectious Diseases, National Institute of Health, KCDC, Heungdeok-gu, Cheongju-si, Chungcheongbuk-do, Republic of Korea; b Division of Biosafety Evaluation and Control, National Institute of Health, KCDC, Heungdeok-gu, Cheongju-si, Cheongbuk-do, Republic of Korea

## Abstract

Here, we announce the complete genome sequence of *Salmonella enterica* serovar Enteritidis (*S*. Enteritidis) bacteriophage MA12, a 41-Kb chromosome. The strain can infect both *Campylobacter jejuni* (*C. jejuni*) and *S*. Enteritidis and can be used in phage therapy experiments with poultry and poultry meat.

## GENOME ANNOUNCEMENT

*Campylobacter* spp. and *Salmonella* spp. are common causes of gastrointestinal disease and are typically transmitted by food-products derived from the poultry industry (1, 2). These pathogens can be controlled by specific phages applied throughout the food chain (3).

Bacteriophage MA12 was isolated from a surface water sample using *Salmonella enterica* serovar Enteritidis (*S*. Enteritidis) PT1b as a host, and it was also able to infect *C. jejuni*. The phage was purified using the top agar method and polyethylene glycol (PEG)-precipitation. Morphological analysis by TEM revealed that phage MA12 belongs to the family *Siphoviridae* (4), it has an icosahedral head of 81 nm, and a long tail of 123 nm by 14 nm.

Genomic DNA was concentrated using PEG 8000 precipitation and isolated using the Sambrook’s phenol extraction method (5). The GS FLX Titanium rapid library preparation kit (Roche Diagnostics, Germany) was used for a sequencing library construction by following the manufacturer’s instructions. The sequence reads were generated using GS FLX platform and *de novo* assembled using GS De Novo Assembler (version 2.9). A total of 15,674 reads (6,964,979 bp) were generated and the average read length was 444,366 bp. The sequencing coverage was 169.8×. A total of 15,667 reads were used for *de novo* assembly and 15,209 reads were assembled into a contig after removing singletons and partial reads. After the *de novo* assembly, the physical ends of the genome were not determined (6). The size of the MA12 genome was 41,224 bp and G+C content was 49.88%. When the genome was compared to the reference genomes, *Salmonella* phage Jersey, the nucleotide homology was approximately 47.2%. The nucleotide homology of MA12 was calculated using EMBOSS Stretcher (EMBOSS version 6.5.7.0) (7). Whole-genome based phylogenetic analysis revealed that the closest neighbor of MA12 is *Salmonella* phage wksl3 and *Salmonella* phage SS3e. The three strains, MA12, wksl3, and SS3e were classified into an independent clade set apart from Jerseylikevirus (8). A multiple sequence alignment was performed using MAFFT (version 7.221) (9) and the whole-genome based phylogenetic tree was inferred using RAxML (version 8.2.0) (10) with the Gamma GTR model. The annotation of the genome was performed using the RAST (Rapid Annotation using Subsystem Technology) server (11). From the annotation, 59 protein-coding sequences were identified. Of those protein-coding sequences, 54 matched identified phage genes, including 47 genes involved in phage structure, six genes involved in DNA replication, one gene of late protein, and one homing endonuclease and the others are protein-coding sequences that encoded hypothetical proteins.

### Accession number(s).

The genome sequence of MA12 was deposited in DDBJ/EMBL/GenBank under the accession number KX245013.
